# Expression of Components of the Renin-Angiotensin System by the Putative Stem Cell Population Within WHO Grade I Meningioma

**DOI:** 10.3389/fsurg.2019.00023

**Published:** 2019-05-16

**Authors:** Ganeshwaran Shivapathasundram, Agadha C. Wickremesekera, Helen D. Brasch, Bede van Schaijik, Reginald W. Marsh, Swee T. Tan, Tinte Itinteang

**Affiliations:** ^1^Gillies McIndoe Research Institute, Wellington, New Zealand; ^2^Department of Neurosurgery, Wellington Regional Hospital, Wellington, New Zealand; ^3^Faculty of Medicine, Auckland University, Auckland, New Zealand; ^4^Wellington Regional Plastic, Maxillofacial and Burns Unit, Hutt Hospital, Wellington, New Zealand

**Keywords:** meningioma, renin-angiotensin system, pro(renin) receptor, angiotensin converting enzyme, angiotensin II receptor I, angiotensin II receptor 2, embryonic stem cells

## Abstract

**Aim:** We have recently demonstrated a putative stem cell population within WHO grade I meningioma (MG) that expressed embryonic stem cell (ESC) markers OCT4, NANOG, SOX2, KLF4 and c-MYC, localized to the endothelial and pericyte layers of the microvessels. There is increasing recognition that the renin-angiotensin system (RAS) plays a critical role in stem cell biology and tumorigenesis. This study investigated the expression of components of the RAS: pro-renin receptor (PRR), angiotensin converting enzyme (ACE), angiotensin II receptor 1 (ATIIR1), and angiotensin II receptor 2 (ATIIR2) on the putative stem cell population on the microvessels of WHO grade I MG.

**Methods:** 3,3-Diaminobenzidine (DAB) immunohistochemical (IHC) staining was performed on WHO grade I MG tissue samples from 11 patients for PRR, ACE, ATIIR1, and ATIIR2. Two of the MG samples subjected to DAB IHC staining underwent immunofluorescence (IF) IHC staining to investigate co-expression of each of these components of the RAS in using combinations of CD34 and ESC marker SOX2 or OCT4. NanoString mRNA expression analysis and Western blotting (WB), were performed on six snap-frozen MG tissue samples to confirm mRNA and protein expression of these proteins, respectively.

**Results:** DAB IHC staining demonstrated expression of PRR, ACE, ATIIR1, and ATIIR2 within all 11 MG tissue samples. WB and NanoString mRNA analyses, confirmed protein and mRNA expression of these proteins, respectively. IF IHC staining showed PRR, ATIIR1 and ATIIR2 were localized to the OCT4^+^ and SOX2^+^ endothelium and the pericyte layer of MG while ACE was localized to the OCT4^+^ endothelium of the microvesels.

**Conclusion:** The novel finding of the expression of PRR, ACE, ATIIR1, and ATIIR2 on the putative stem cell population on the microvessels of WHO grade I MG, suggests that these stem cells may be a potential therapeutic target by manipulation of the RAS.

## Introduction

Meningioma (MG) is a common primary central nervous system neoplasm accounting for 25–30% of primary intracranial and intraspinal tumors (1). MG is thought to arise from arachnoid cap cells of the brain and spinal cord (2), based on correlational histological, and ultrastructural studies comparing arachnoid cap cells with MG cells (2). Arachnoid cap cells are thought to arise from neural crest neuroectoderm which differentiates from pluripotent stem cells (3).

Tumor stem cells are the suggested cellular origin of cancer including glioblastoma (GB) (4) and leukemia (5) and are increasingly thought to give rise to benign conditions such as Dupuytren's disease (6), infantile hemangioma (IH) (7), and MG (1, 8–10). Cultured MG cells demonstrate tumorsphere formation (9), self-renewal, and expression of embryonic stem cell (ESC) associated markers including SOX2, nestin (1), and KLF4 (11). We have previously identified and characterized a putative stem cell population, localized to both the endothelial and the pericyte layers of the microvessels of WHO grade I MG (12).

The renin-angiotensin system (RAS) is an endocrine system known for the maintenance of blood pressure and electrolyte homeostasis. Renin is physiologically secreted from the juxtaglomerular apparatus in response to reduced arterial pressure, decreased sodium in the distal tubule or sympathetic nervous system activity via ß-adrenergic receptors (13). Renin converts angiotensinogen to angiotensin I (ATI) which is then converted to angiotensin II (ATII) by angiotensin converting enzyme (ACE) (14). ATII binds to ATII receptor 1 (ATIIR1) and ATII receptor 2 (ATIIR2) (14). The expression of components of the RAS on the tumor stem cells in a number of cancer types including oral cavity squamous cell carcinoma (15) and GB (16) implies a crucial role for the RAS in the maintenance and regulation of their behavior.

In 1998 Lever et al. (17), in a retrospective cohort study, show a reduced relative risk of cancer in patients administered ACE inhibitors. Since then the role of the RAS in other cancer has been investigated (18–23). Dysfunction of the RAS may play an important role in carcinogenesis. Therefore, identifying expression of components of RAS by MG stem cells may result in the development of novel treatment, especially for the more difficult, aggressive, recurrent, anaplastic, or skull base, MGs.

This study was aimed at investigating the expression of components of RAS, in relation to the putative stem cell population on the microvessels of WHO grade I MG, using immunohistochemical (IHC) staining, Western blotting (WB), and NanoString mRNA analysis.

## Materials and Methods

### Tissue Samples

WHO grade I MG from one male and 10 female patients, aged 36–85 (mean, 61.8) years, included in our previous study (12), were obtained from the Gillies McIndoe Research Institute Tissue Bank for this study which was approved by the Central Region Health and Disability Ethics Committee (ref. no. 15/CEN/28/AM01) with written informed consent from all patients.

### Immunohiostochemical Staining

Four micrometer thick formalin-fixed paraffin-embedded sections of WHO grade I MG samples from 11 patients were subjected to 3,3-diaminobenzidine (DAB) IHC staining for (pro)renin receptor (PRR) (1:2000; cat# ab40790, Abcam, Cambridge, UK), ACE (1:100; cat# MCA2054, AbD Serotec, Kidlington, UK), ATIIR1 (1:30; cat# ab9391, Abcam and ATIIR2 (1:2000; cat# NBP1-77368, Novus Biologicals, LLC, Littleton, CO, USA). Surgipath Micromount (Leica) was used to mount all the slides. Staining of MG sections with a mouse (ready-to-use; cat# IR750, Dako, Copenhagen, Denmark) and rabbit (ready-to-use; cat# IR600, Dako) primary antibody isotype control combination was performed as an appropriate negative control, as previously described (24).

Two of the MG samples subjected to DAB IHC staining underwent immunofluorescence (IF) IHC staining using combinations of CD34 (ready-to-use; cat# PA0212, Leica), ERG (ready-to-use; cat# EP111, Cell Marque, Rocklin, CA, USA) (25), and OCT4 (1:30; cat# MRQ-10, Cell Marque) that highlighted the endothelium and stem cells on the microvessels, respectively (13). Appropriate positive human control tissues included placenta for PRR, liver for ACE and ATIIR1, and kidney for ATIIR2.

### NanoString mRNA Analysis

RNA extracted from six snap-frozen MG samples of the original cohort of 11 patients included in DAB IHC staining was subjected to NanoString mRNA analysis (NanoString Technologies, Seattle, WA, USA) for mRNA transcripts for PRR (ATP6AP2, NM_005765.2), ACE (NM_000789.2), ATIIR1 (AGTR1, NM_000685.3), ATIIR2 (AGTR2, NM_000686.3), and the housekeeping gene GUSB (NM_000181.1), performed by New Zealand Genomics (Dunedin, New Zealand).

### Image Capture

DAB IHC-stained images were viewed and captured on an Olympus BX53 microscope (Tokyo, Japan) and Cellsens 2.0 software (Olympus). IF IHC-stained images were captured on an Olympus FV1200 biological confocal laser-scanning microscope with subsequent 2D deconvolution using cellSens Dimension 1.11 software (Olympus).

### Western Blotting

Total protein was extracted from six snap-frozen MG samples of the original cohort of 11 patients used for DAB IHC staining, separated by SDS-PAGE, and transferred to a PVDF membrane using methods previously described (16). Detection of the proteins was performed on the iBind Flex (cat# SLF2000, Thermo Fisher Scientific) using the primary antibodies for PRR (ATP6IP2, 1:500, cat# ab40790, Abcam), ATIIR1 (1:500; cat# sc-1173, Santa Cruz, CA, USA), ATIIR2 (1:1000; cat# ab92445, Abcam), ACE (1:200; cat# sc-12184, Santa Cruz), and α-tubulin (1:1000; cat# 62204, Thermo Fisher Scientific). Appropriate secondary antibodies were goat anti-rabbit Alexa Fluor 647 (1:2000; cat# A21244, Life Technologies), chicken anti-goat Alexa Fluor 647 (1:2000; cat# A21469, Life Technologies) and goat anti-mouse Alexa Fluor 488 (1:2000; cat# A21202, Life Technologies). The ChemiDoc MP Imaging System (Bio-Rad) and Image Lab 5.0 software (Bio-Rad) were used for band detection and analysis. All experiments were performed in triplicate. Snap-frozen human placenta tissue was used as positive control for PRR and ATIIR1, snap-frozen mouse lung tissue was used as positive control for ACE, and a recombinant ATIIR2 protein (cat# H00000186-P01, Novus Biologicals) was used as an appropriate positive control. Matched mouse (1:500; cat# ab18443, Abcam) and rabbit (1:500; cat# ab171870, Abcam) isotype controls were used as appropriate negative controls.

### Statistical Analysis

Statistical analysis of the NanoString mRNA data was performed using the *t*-test (SPSS v24).

## Results

### DAB IHC Staining

**Figure 1:**
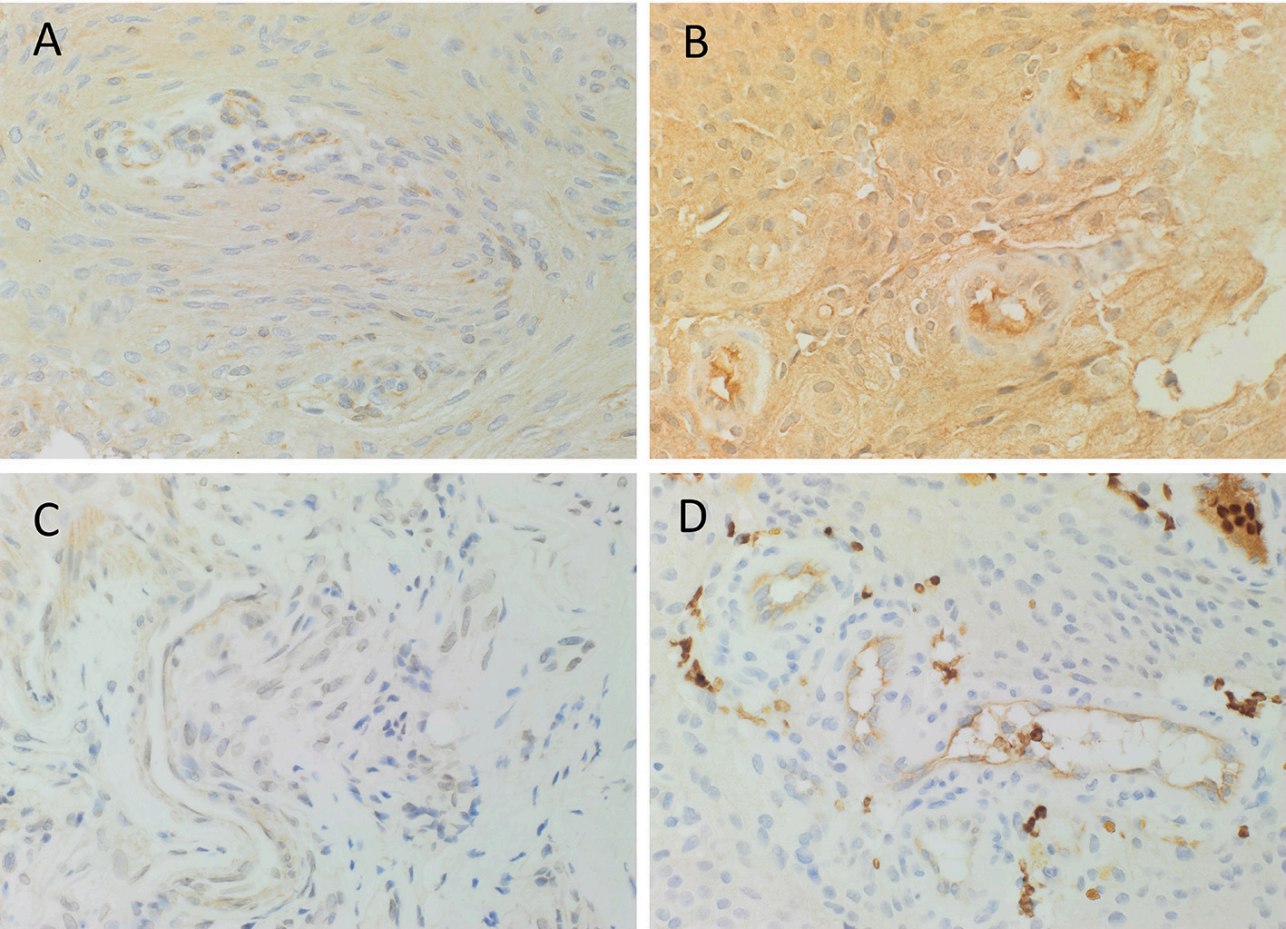
Representative 3,3-diaminobenzidine immunohistochemical-stained images demonstrating the expression of PRR (**A**, brown), ATIIR1 (**B**, brown), and ATIIR2 (**C**, brown) on both the endothelial and pericyte layers of the microvessels of WHO grade I meningioma. ACE (**D**, brown) was expressed only on the endothelium of the microvessels. Nuclei were counterstained with hematoxylin (**A–D**, blue). Original magnification: 400X.

DAB IHC staining demonstrated abundant expression of PRR (Figure 1A, brown), ATIIR1 (Figure 1B, brown), and ATIIR2 (Figure 1C, brown) on both the endothelial and pericyte layers of the microvessels of the MG tissue samples examined. ACE (Figure 1D, brown) was expressed only on the endothelium of the same microvessels.

The positive controls for PRR (Supplementary Figure 1A, brown) in placenta, ATIIR1 (Supplementary Figure 1B, brown) in liver, ATIIR2 (Supplementary Figure 1C, brown) in kidney, and ACE (Supplementary Figure 1D, brown) in liver, demonstrated the expected staining patterns. The negative controls (Supplementary Figure 1E) demonstrated no staining.

### IF IHC Staining

**Figure 2:**
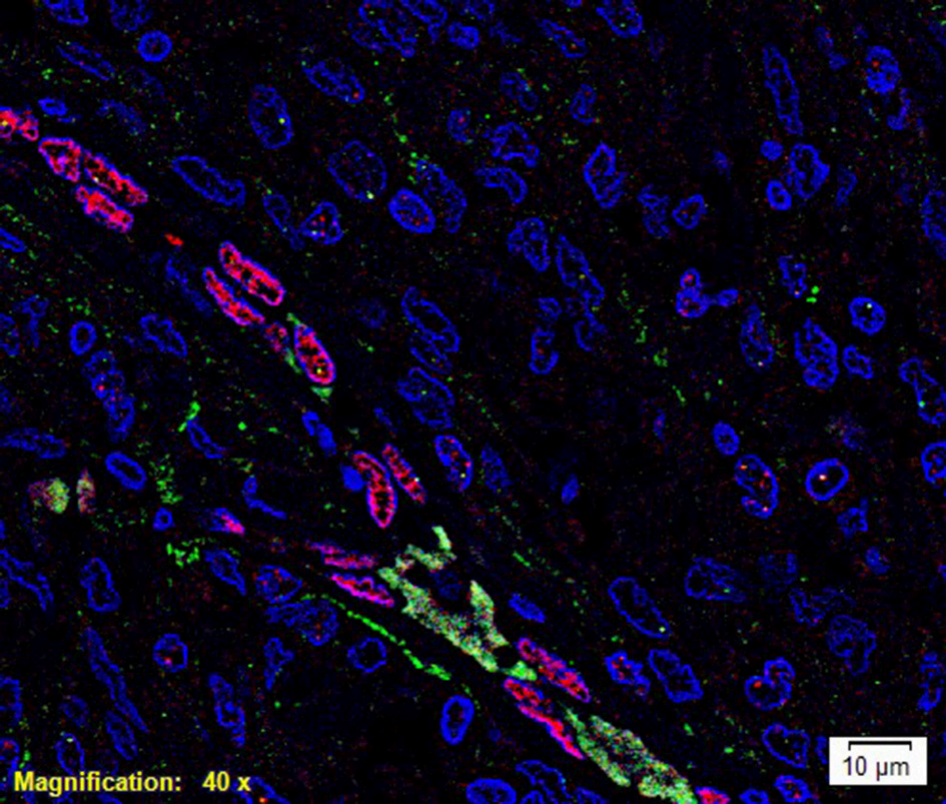
A representative immunofluorescence immunohistochemical- stained image of WHO grade I meningioma demonstrating the expression of OCT4 (green) on the ERG^+^ (red) endothelium and the pericyte layer of the microvessels. Cell nuclei were counterstained with 4′, 6′-diamidino-2-phenylindole (blue). Scale bar: 10 μm.

**Figure 3:**
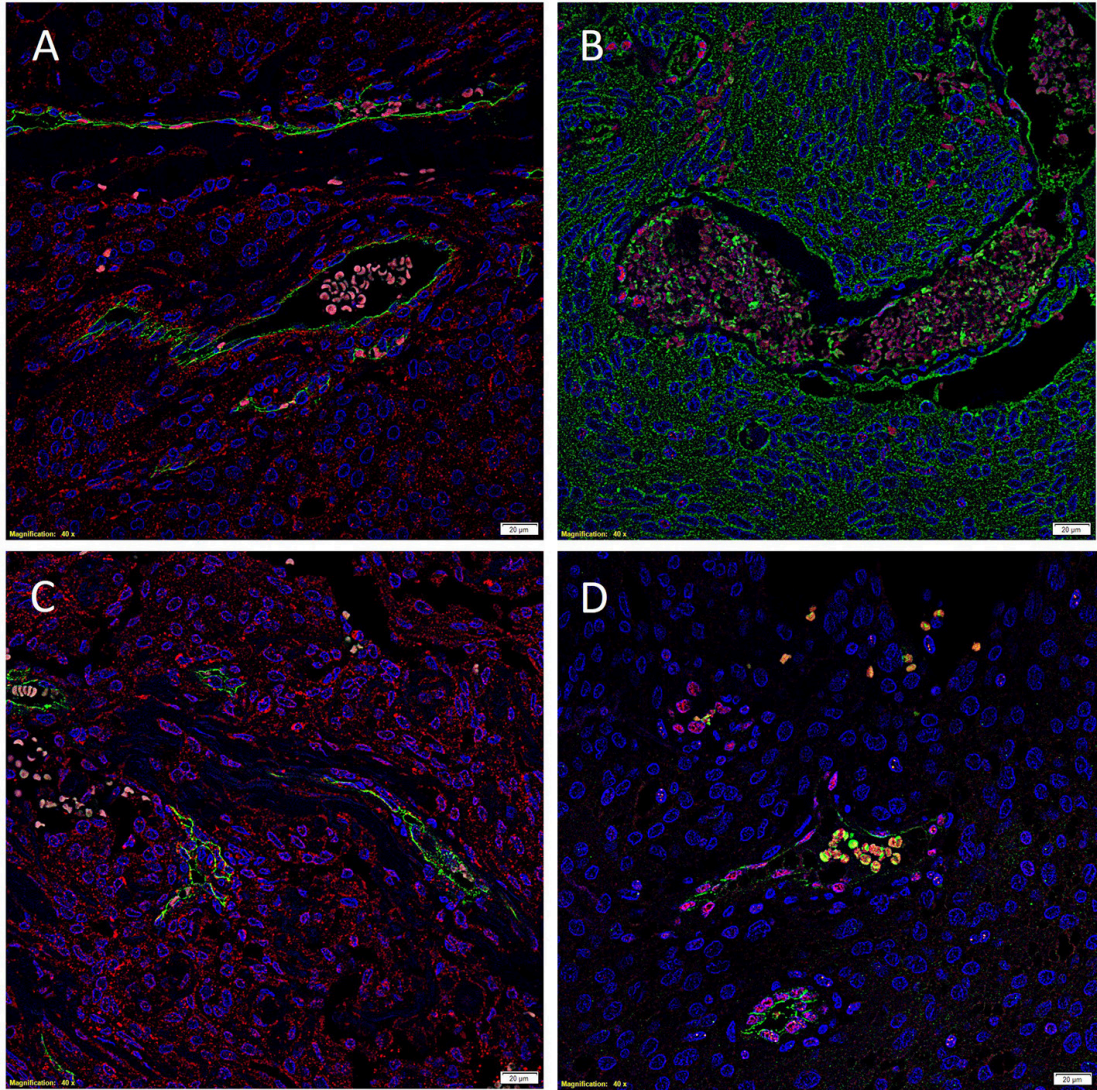
Representative immunofluorescence immunohistochemical-stained images of WHO grade I meningioma demonstrating the expression of PRR (**A**, red), ATIIR1 (**B**, green), and ATIIR2 (**C**, red) on the CD34^+^ (**A,C**, green) and ERG^+^ (**B**, red) endothelium and the outer pericyte layer. ACE (**D**, green) was only expressed by the CD34^+^ (**D**, red) endothelial layer. Cell nuclei were counterstained with 4′, 6′-diamidino-2-phenylindole (**A–D**, blue). Scale bars: 20 μm.

To investigate localization of components of the RAS, co-staining was performed with either CD34 or ERG (25), markers for the endothelium. ESC marker OCT4 was used to highlight the putative stem cell population on the endothelial and pericyte layers of the microvessels in WHO grade I MG we have previously identified (12). OCT4 (Figure 2, green) was expressed on both the ERG^+^ (Figure 2, red) endothelium as well as the outer pericyte layer. PRR (Figure 3A red), ATIIR1 (Figure 3B, green) and ATIIR2 (Figure 3C, red) were expressed by the CD34^+^ (Figures 3A,C) and ERG^+^ (Figure 3B) endothelium as well as the outer pericyte layer. ACE (Figure 3D, green) was only expressed by the ERG^+^ (Figure 3D, red) endothelium endothelial layer.

Images illustrating the individual stains demonstrated in Figure 3 are presented in Supplementary Figure 2. Negative isotype controls, demonstrated minimal staining as expected (Supplementary Figure 2I).

### Nanostring mRNA Analysis

**Figure 4:**
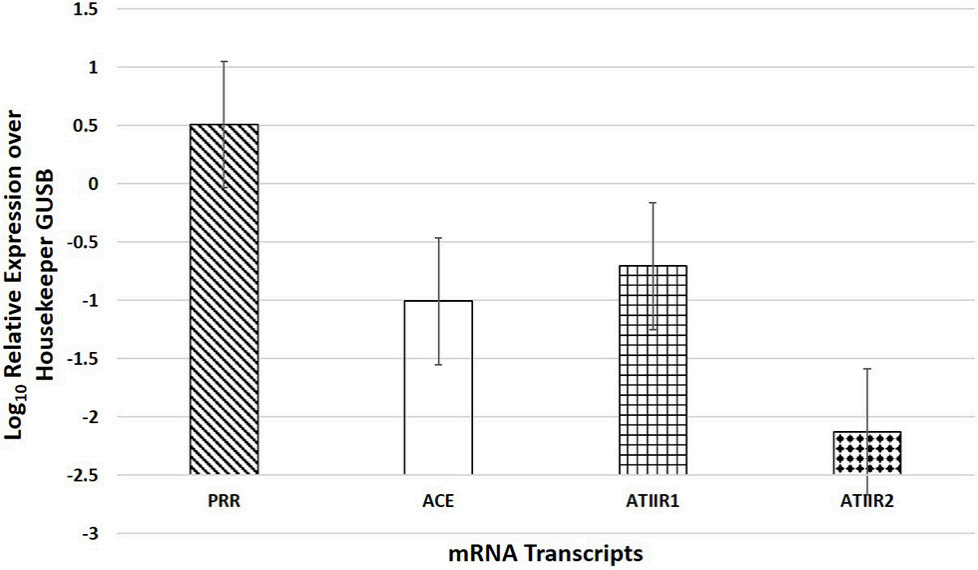
NanoString mRNA analysis of six WHO grade I meningioma samples demonstrating the presence of mRNA transcripts for PRR, ACE, ATIIR1, and ATIIR2 normalized against the housekeeper gene GUSB.

NanoString mRNA analysis demonstrated mRNA transcripts for all four components of the RAS investigated (*p* < 0.05), and the expression of PRR was significantly higher than ACE (*p* = 0.004) and ATIIR1 (*p* = 0.007), while ACE was not significantly different from ATIIR1 (*p* = 0.681) (Figure 4).

### Western Blotting

**Figure 5:**
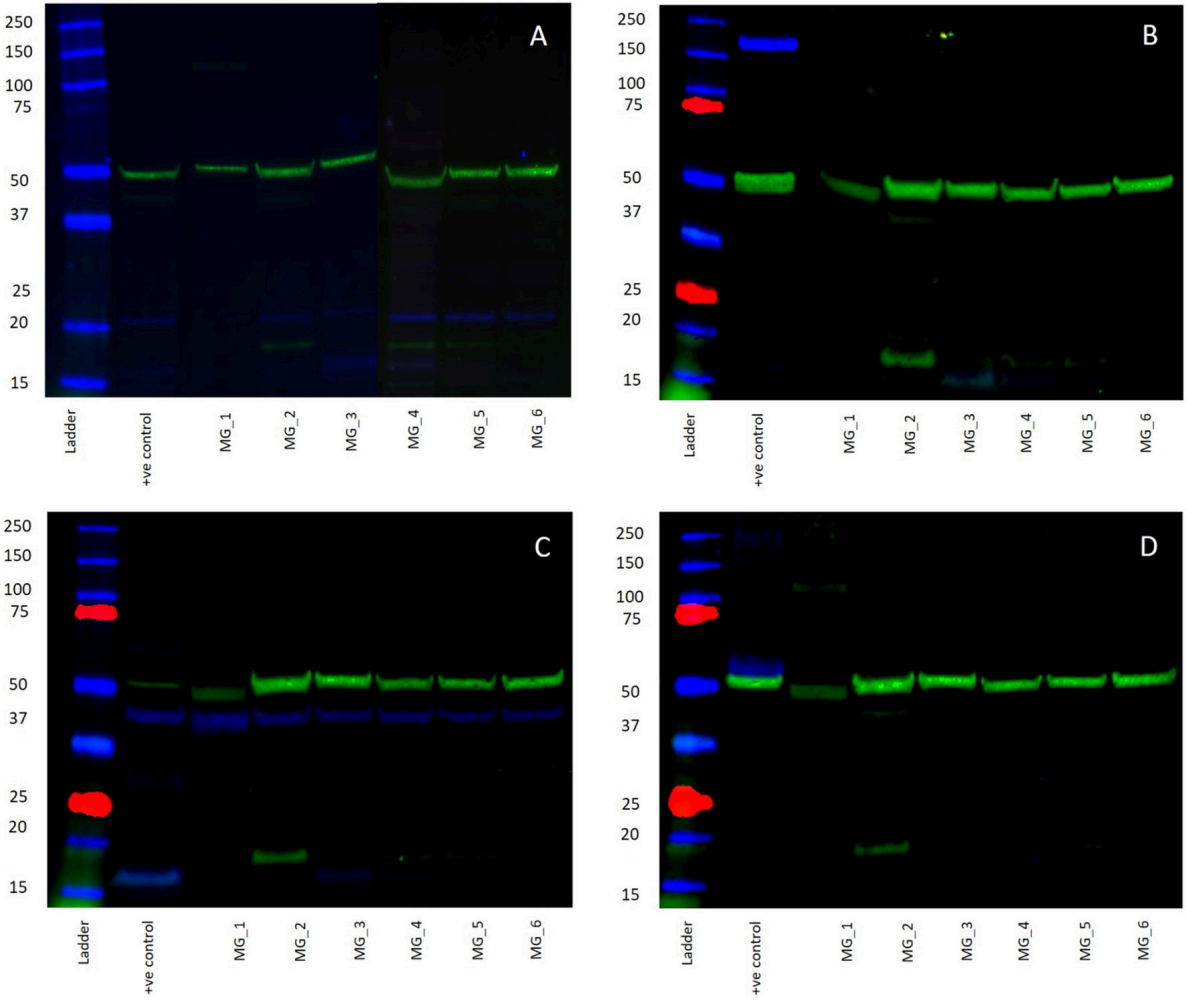
Western blots demonstrating the expression of PRR at 21 kDA (**A**, blue) and ATIIR1 at 42 kDa (**C**, blue), but not ACE (**B**, blue), or ATIIR2 (**D**, blue). Bands for α-tubulin (**A–D**, green) confirmed efficient transfer and approximate equivalent protein loading for all WHO grade I meningioma samples examined.

WB of the six snap-frozen MG samples demonstrated the presence of bands at the expected molecular weight for PRR and ATIIR1 but not ACE or ATIIR2. PRR was detected in five out of six MG tissue samples (Figure 5A, blue) at ~21 kDa. ATIIR1 was detected in all MG tissue samples (Figure 5C, blue) with bands at the expected molecular weight of 42 kDa (2). ACE and ATIIR2 were below detectable levels in all MG tissue samples (Figures 5B,D, blue) investigated. Bands for α-tubulin (Figures 5A–D, green) confirmed efficient transfer and approximate equivalent protein loading for all samples examined. The respective positive controls used confirmed specificity of the antibody for their target protein. The rabbit and mouse IgG isotype controls (Supplementary Figure 3) were used to detect any non-specific staining and therefore confirmed the presence of the components of the RAS.

## Discussion

The tumor stem cell concept is novel especially pertaining to benign tumors such as MG. There is an increasing body of evidence supporting the presence of tumor stem cells within MG (1, 8, 9, 11, 26). We have demonstrated that this primitive population within MG is localized to both the endothelial and pericyte layers of the microvessels within the tumor by its expression of the ESC markers including OCT4 and NANOG (12).

The RAS is an endocrine system known for its physiological role in blood pressure regulation and electrolyte homeostasis (14). Components of the RAS play important roles in angiogenesis, cellular proliferation, and apoptosis (19, 20). ATII is the primary effector of the RAS (19), mediated via its two receptors ATIIR1 and ATIIR2 (19). ATII induces phosphorylation of mitogen-activated protein kinase in prostate cancer cells and also induces VEGF which is important in tumor angiogenesis (19, 27).

The two ATII receptors have opposing functions. ATIIR1 induces angiogenesis and cellular proliferation, while ATIIR2 functionally antagonizes these actions (27, 28). ATIIR1 is overexpressed in breast cancer (18) and is associated with tumor invasiveness in ovarian (22) and cervical (18, 20) cancer. In contrast, ATIIR2 induces apoptosis of cancer cells and inhibits cellular proliferation (14, 20).

PRR is another important component of the RAS implicated in carcinogenesis through its involvement in the Wnt/ß-catenin signaling pathway (14). Wnt/ß-catenin signaling is important in carcinogeneis and embryonic development (29). PRR is essential in normal Wnt/ß-catenin signaling, with overexpression of PRR resulting in a loss of control of cell proliferation (14). Overexpression of PRR has been identified in the development of glioma via activation of the Wnt/ß-catenin signaling pathway (30).

We have demonstrated the presence of components of the RAS within the putative stem cell population in WHO grade I MG with PRR being the most abundantly expressed at both the protein and mRNA levels, by IHC staining, WB and NanoString mRNA analyses. This was in contrast to ACE and ATIIR2, which were only detected by IHC staining and NanoString mRNA analysis, but was below detectable levels by WB. This may, in-part, be accounted for by low levels of the protein relative to the whole proteome content of the tissues. Components of the RAS have been recently identified in benign conditions including Dupuytren's disease (31) and IH (32). In the case of IH this discovery underscores the novel therapy using β-blockers (33) and ACE inhibitors (34).

This is the first report demonstrating the expression of PRR, ACE, ATIIR1, and ATIIR2 on the microvessels of WHO grade I MG, on which we have recently demonstrated the presence of a putative stem cell population. It is exciting to speculate that the peptides of the RAS are essential to the survival of these tumor stem cells which may be a novel therapeutic target by modulation of the RAS.

This study provides novel insights into the biology of MG, however, larger studies including *in vitro* and *in vivo* experiments are required to validate the true functional role of the RAS on the tumor stem cells in MG.

## Conclusion

We have demonstrated the presence of components of the RAS: PRR, ACE, ATIIR1, and ATIIR2, localized to the putative stem cell population on the microvessels of grade I MG. These results support the hypothesis that this primitive population, the proposed origin of MG, may be potentially targeted by modulating the RAS.

## Ethics Statement

This study was approved by the Central Region Health and Disability Ethics Committee (Ref. no. 15/CEN/28/AM01).

## Author Contributions

TI and ST formulated the study hypothesis. TI, AW, and ST designed the study and interpreted the IF IHC data. TI, HB, AW, GS, and ST interpreted the DAB IHC data. BvS performed WB analysis. BvS, TI, AW, and ST interpreted the WB data. TI and ST interpreted the NanString data. RM performed the statistical analysis. GS, AW, TI, and ST drafted the manuscript. All authors commented on and approved the manuscript.

### Conflict of Interest Statement

TI and ST are inventors of the PCT patents Cancer Diagnosis and Therapy (PCT/NZ2015/050108) and Cancer Therapeutic (PCT/NZ2018/050006), and provisional patent application Novel Pharmaceutical Compositions for Cancer Therapy (US/62/711709). The remaining authors declare that the research was conducted in the absence of any commercial or financial relationships that could be construed as a potential conflict of interest.

## Supplementary Material

Supplementary Figure 1 3,3-Diaminobenzidine immunohistochemical-stained sections of placenta for PRR (**A**, brown), liver for ATIIR1 (**B**, brown), kidney for ATIIR2 (**C**, brown), and ACE (**D**, brown), and staining with an IgG isotype antibody provided an appropriate negative control **(E)**. Nuclei were counterstained with hematoxylin (**A–E**, blue). Original magnification: 400X.

Supplementary Figure 2 Split images of immunofluorescence immunohistochemical-stained sections of WHO grade I meningioma shown in Figure 3 demonstrating the expression of PRR (**A**, red) and CD34 (**B**, green), ATIIR1 (**C**, red) and CD34 (**D**, green), ATIIR2 (**E**, red) and CD34 (**F**, green), ERG (**G**, red) and ACE (**H**, green). A negative control (**I**) to test the specificity of the fluorescent secondary antibodies is performed on a section of MG. Cell nuclei were counterstained with 4′,6-diamidino-2-phenylindole (**A–I**, blue). Scale bars: 20 μm.

Supplementary Figure 3 Western Blot image of rabbit and mouse IgG isotype controls used to detect any non-specific staining and therefore confirmed the presence of the components of the renin-angiotensin system.
