# Supplementary figures and images for: Expression of Components of the Renin-Angiotensin System by the Putative Stem Cell Population Within WHO Grade I Meningioma
Source: Front Surg. 2019 May 16;6:23. doi: 10.3389/fsurg.2019.00023 (PMC6532688; doi:10.3389/fsurg.2019.00023)

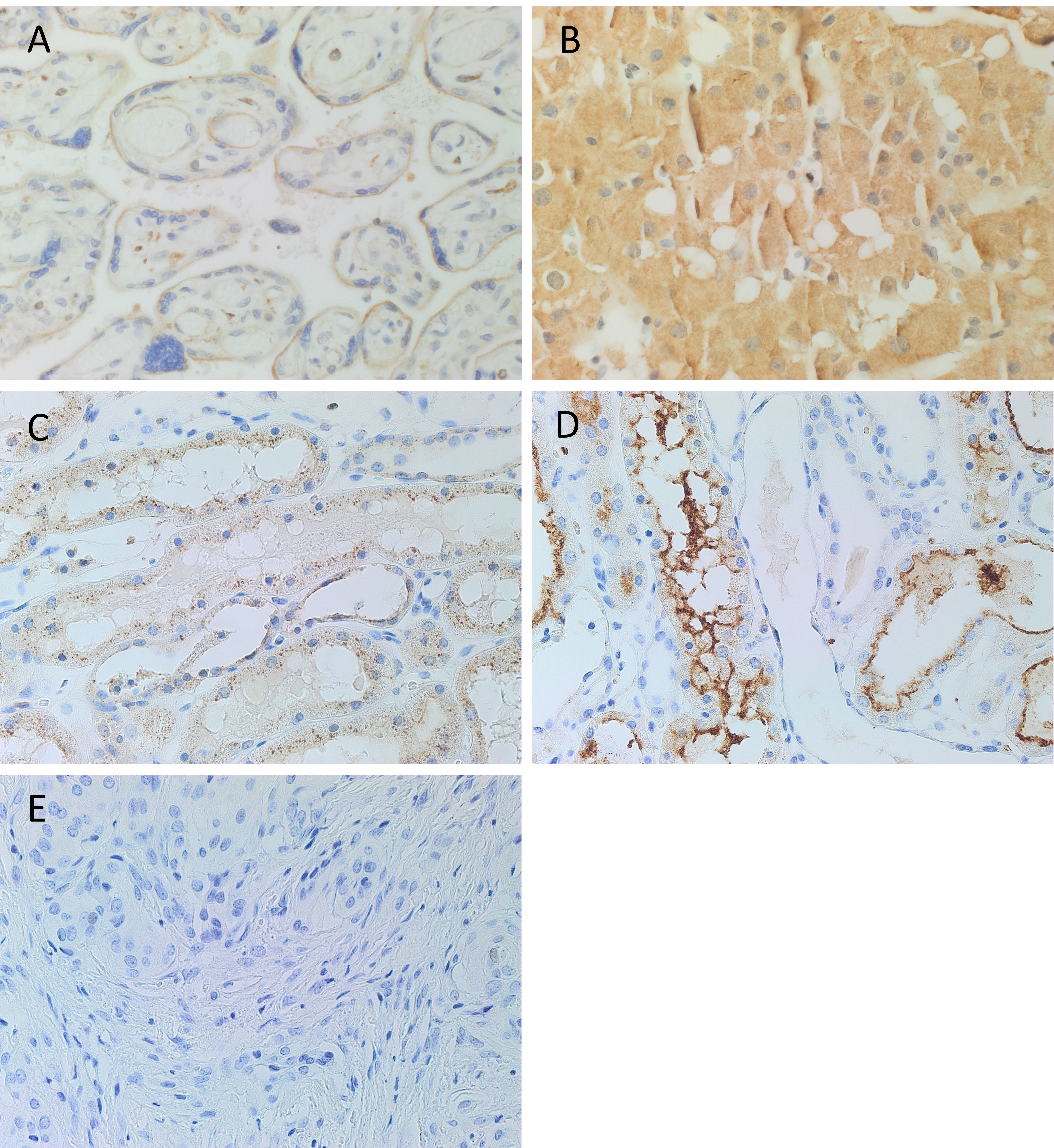

Supplement: Supplementary Figure 1 — 3,3-Diaminobenzidine immunohistochemical-stained sections of placenta for PRR (A, brown), liver for ATIIR1 (B, brown), kidney for ATIIR2 (C, brown), and ACE (D, brown), and staining with an IgG isotype antibody provided an appropriate negative control (E). Nuclei were counterstained with hematoxylin (A–E, blue). Original magnification: 400X. [file Image_1.JPEG]

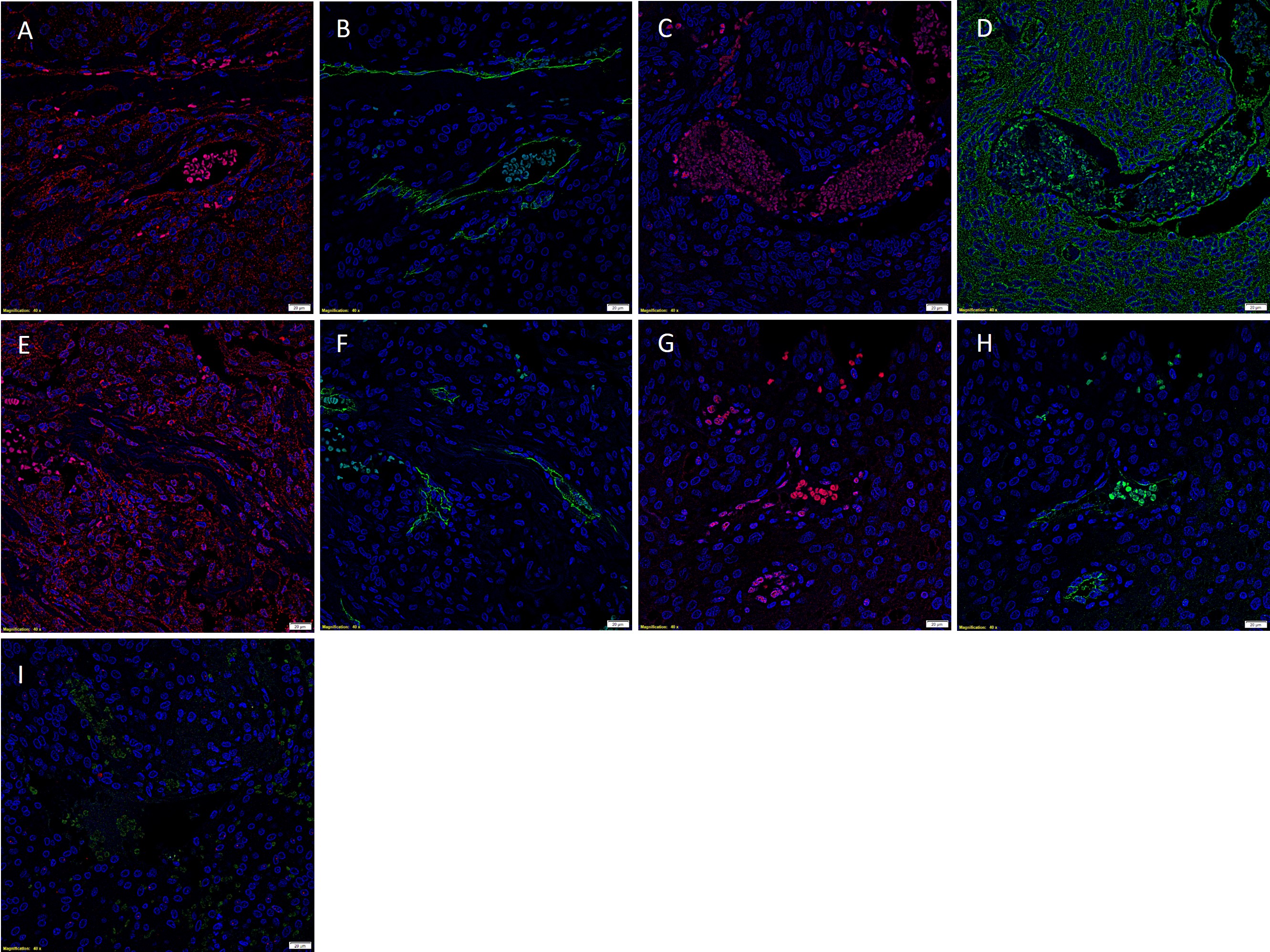

Supplement: Supplementary Figure 2 — Split images of immunofluorescence immunohistochemical-stained sections of WHO grade I meningioma shown in Figure 3 demonstrating the expression of PRR (A, red) and CD34 (B, green), ATIIR1 (C, red) and CD34 (D, green), ATIIR2 (E, red) and CD34 (F, green), ERG (G, red) and ACE (H, green). A negative control (I) to test the specificity of the fluorescent secondary antibodies is performed on a section of MG. Cell nuclei were counterstained with 4′,6-diamidino-2-phenylindole (A–I, blue). Scale bars: 20 μm. [file Image_2.JPEG]

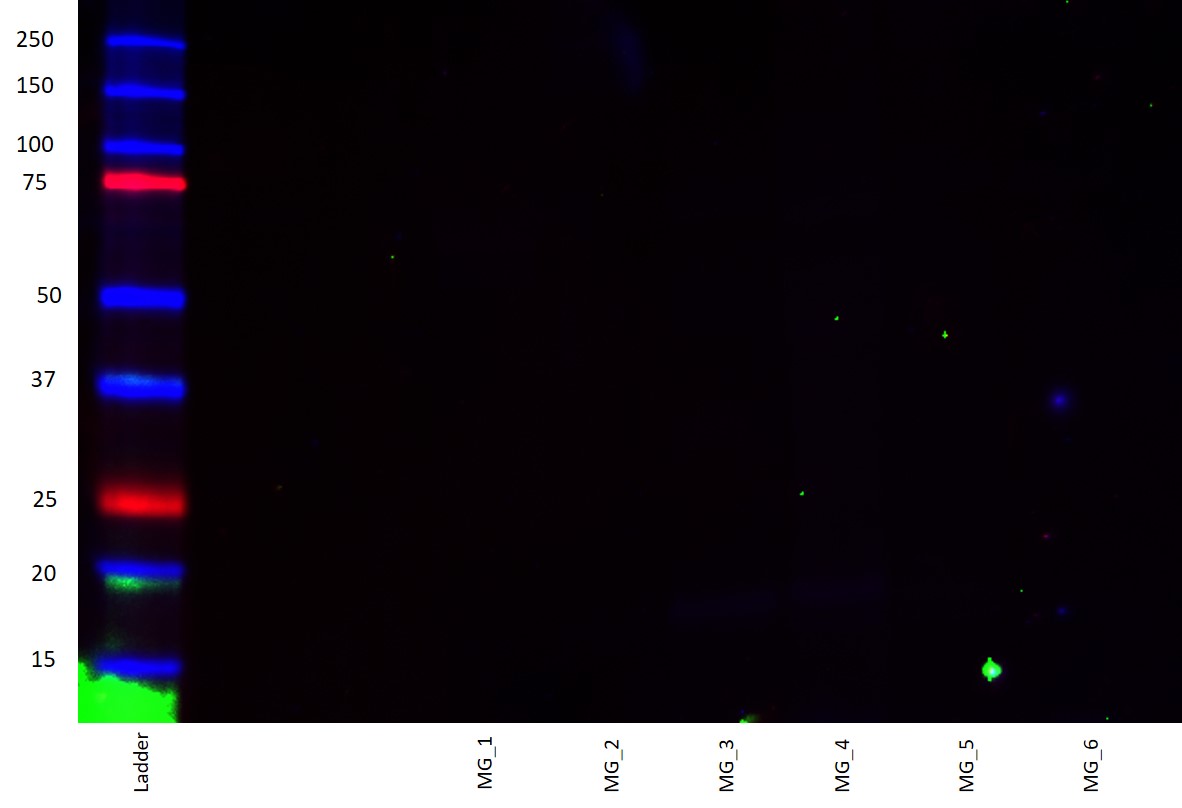

Supplement: Supplementary Figure 3 — Western Blot image of rabbit and mouse IgG isotype controls used to detect any non-specific staining and therefore confirmed the presence of the components of the renin-angiotensin system. [file Image_3.JPEG]
